# College and Hospital Note

**Published:** 1897-03

**Authors:** 


### College and Hospital Note.

THE NEW YORK POST-GRADUATE MEDICAL SCHOOL AND HOSPITAL. —An interesting correspondence has lately been published in the *Medical Record* concerning this institution, which want of space prevents us from quoting in full. It would appear that an attempt has been made to prevent the Post-Graduate from obtaining the usual appropriation from the board of estimates and apportionment. Dr. D. B. St. John Roosa, president of the Post-Graduate School and Hospital, makes conclusive answer in the *Medical Record*, January 30, 1897, to any objections that may have been raised to the appropriation. In this letter it is shown that the school over which Dr. Roosa so appropriately and ably presides is doing a great charity work, and is in the highest sense a charitable organisation. There is no reason why discrimination should be made in these appropriations. The Post-Graduate is most assuredly entitled to its proportion of the annual sum allotted to similar charities.

